# Friends and foes in the obligate plant pathobiome

**DOI:** 10.1371/journal.ppat.1013756

**Published:** 2025-12-11

**Authors:** Yiheng Hu, Marion Müller, Thomas Lahaye, Ralph Hückelhoven, Eric Kemen

**Affiliations:** 1 Centre of Plant Molecular Biology (ZMBP), Eberhard-Karls-University of Tübingen, Tübingen, Germany; 2 Chair of Phytopathology, TUM School of Life Sciences, Technical University of Munich, Freising-Weihenstephan, Germany; University of Tübingen: Eberhard Karls Universitat Tubingen, GERMANY

Obligate plant pathogens are a group of microbes that depend entirely on living host tissue for survival and reproduction. This biotrophic lifestyle, exemplified by cereal rusts, powdery and downy mildews, and white blister rusts, enables nutrient uptake without killing host cells. These pathogens form intimate feeding interfaces such as haustoria and have undergone extensive gene loss, including in primary metabolism, reflecting their profound host dependence [1]. Their inability to grow in axenic culture highlights this evolutionary specialisation.

Plant diseases have long been viewed through a binary lens of host and pathogen, a framework formalised in the classical disease triangle, where disease outcomes emerge from the interplay among the host, pathogen, and environment. Environmental factors such as leaf wetness, nutrient availability, and abiotic stresses like drought or heat can shift both pathogen success and microbial community composition, thereby influencing disease outcomes. For example, high humidity can dramatically enhance rust infection [2]. Yet, this model overlooks another key determinant of plant health: the microbiome. Plants exist not as isolated individuals but as holobionts—complex assemblages of the host and its associated microbial community [3]. It is increasingly evident that these resident microbes influence pathogen succeed [4], adding a fourth dimension to the disease triangle and transforming it into a disease diamond (Fig 1).

**Fig 1:**
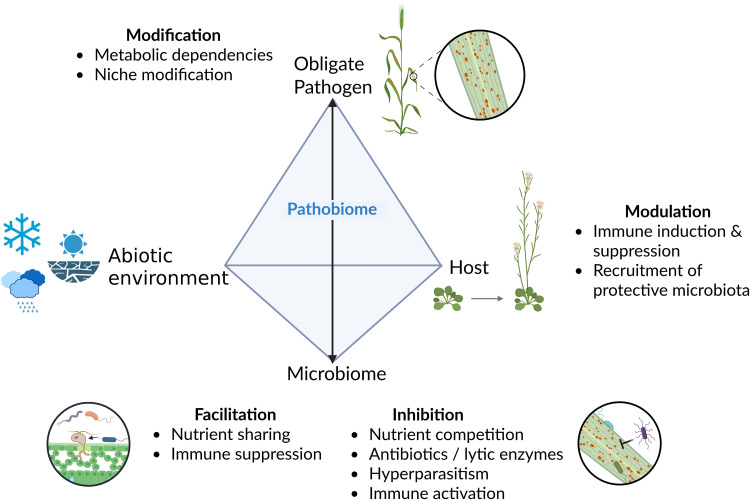
Expanded disease diamond illustrating the central role of the obligate pathobiome in shaping pathogen success. Traditional models of plant disease focus on three primary components: the host, the pathogen, and the environment. For obligate plant pathogens, however, the microbiome—specifically the surrounding microbial community—acts as a critical fourth dimension. This “pathobiome” can influence pathogenicity in multiple ways: (1) Suppressing infection through competition, antibiosis, or hyperparasitism; (2) Facilitating infection via mechanisms such as nutrient cross-feeding or immune suppression; and (3) Interacting with host responses to modulate defence outcomes. The figure was created in BioRender. Kemen, E. (2025) https://BioRender.com/y8and0n.

This broader context is particularly relevant to obligate pathogens, whose entire life cycle unfolds on living plants over extended periods [5]. Infection by obligate pathogens takes place within the pathobiome, comprising the pathogen, the host, and the surrounding microbial community that collectively influence disease outcomes [6,7]. Commensal microbes may compete with pathogens for nutrients and space, produce inhibitory compounds, or prime host defences, thereby acting as microbial antagonists. Conversely, other microbes can promote infection by supplying essential metabolites, altering local environments, or dampening host immunity. For obligate biotrophs with reduced metabolic autonomy, these relationships can be decisive, tipping the balance between successful colonisation and failure. In this Pearl, we explore how microbial communities interact with obligate pathogens as both friends and foes, and how the plant actively modulates these interactions within the pathobiome context.

## How do microbes suppress obligate pathogens?

Within the pathobiome, many plant-associated microbes act as natural antagonists of obligate pathogens. They can inhibit pathogen growth directly by producing antimicrobial compounds, competing for nutrients and space, or through hyperparasitism.

In model systems, an epiphytic yeast *Moesziomyces bullatus* isolated from wild *Arabidopsis thaliana* reduces infection by the white rust pathogen *Albugo laibachii* by secreting a GH25 hydrolase that inhibits *Albugo* colonisation [8]. This enzyme was further demonstrated to mediate cross-kingdom microbial competition in the leaf [9]. This striking example illustrates how a single microbial enzyme can interfere with pathogen infection.

The leaf surface is a nutrient-poor environment where fast-growing commensals can outcompete pathogens for available resources such as sugars, amino acids, and iron [10,11]. For example, *Sphingomonas* bacteria efficiently metabolise diverse carbon sources and secrete secondary metabolites, limiting pathogen access to nutrients and in parallel changing habitat conditions significantly [10]. In addition, high-affinity siderophores produced by certain bacteria can sequester iron, creating conditions that are unfavourable to obligate fungi [12]. Beyond nutrient competition, commensals can also restrict pathogen colonisation through physical exclusion. By pre-emptively occupying key entry points such as stomata or trichomes, fast-growing commensals reduce opportunities for pathogen entry [13]. Furthermore, some phyllosphere bacteria form dense, structured biofilms that act as physical barriers, preventing spore attachment or appressorium formation by fungal pathogens [14].

Understanding microbe-mediated pathogen suppression has led to innovations in biological control strategies in various obligate crop pathogens. The biocontrol of powdery mildew is one of the best-studied systems, with nearly every type of negative microbial interaction was observed. Biocontrol agents such as *Ampelomyces*, *Bacillus*, and *Moesziomyces* have proven effective against multiple powdery mildew species on horticultural crops through niche competition [15], secretion of antimicrobial compounds [16], and parasitism of diverse powdery mildew structures [17]. A few filamentous fungi have also been identified to inhibit wheat rust infection, likely through direct parasitism [18,19].

Overall, microbial communities act as a natural check on obligate plant pathogens. By killing, parasitising, or outcompeting the pathogen, they can effectively restrict infection of obligate pathogens. Studies have shown that resistant or healthy plants often harbour more complex microbial networks than diseased ones, suggesting that microbiome diversity itself contributes to disease suppression, very much reminiscent of general ecosystems’ stability dependence on biodiversity [10]. Therefore, understanding the biotic interactions of obligate pathogens within microbial communities is a promising approach for developing biocontrol strategies and managing plant diseases.

## How can some microbes mutualistically interact with obligate pathogens?

While many microbes hinder obligate pathogens, others can promote their infection or persistence. These mutualistic interactions are often subtle and context-dependent, involving metabolic exchange, immune interference, or environmental modification that collectively shape disease outcomes

One form of facilitation is nutrient cross-feeding where an organism obtains essential nutrients from its host or associated microbes. This reliance has shaped the genomes of obligate biotrophs leading to the loss of key metabolic pathways that are compensated for by their partners. For example, both powdery mildew fungi and the oomycete genus *Albugo* are auxotrophic for thiamine (vitamin B₁) and must acquire it from external sources [20,21]. While the host provides some nutrients, recent findings show that commensal microbes can also supply essential compounds. Hu and colleagues [22] demonstrated that phyllosphere basidiomycete yeast taxa can provide thiamine, thereby promoting the growth of co-occurring obligate pathogens. In this way, the microbial network generates a metabolic niche that compensates for the metabolic limitations of the pathogen. Understanding such metabolic dependencies could potentially offer new approaches for controlling pathogenic microbes.

Microbial interactions that enhance infection are not limited to commensals, mutualists can also promote pathogen infection. Arbuscular mycorrhizal fungi, while generally beneficial, were shown to increase host susceptibility to the obligate fungus *Erysiphe pisi* in *Astragalus* [23]. This suggests that accommodation of mutualists can divert host immune resources, indirectly benefiting foliar pathogens.

Importantly, facilitation is often incidental rather than cooperative, as microbes may unintentionally alter the host environment. For example, bacteria that produce auxin to support their own colonisation may inadvertently influence host signalling pathways, resulting in reduced resistance. Similarly, biofilm-forming epiphytes can increase leaf moisture retention, creating microenvironments favourable for spore germination [24]. In a related process, some phylloplane yeasts secrete esterases that degrade the cuticle, potentially weakening the plant’s first physical barrier to infection and further increasing surface humidity [25].

Within the disease diamond framework, these examples underscore that facilitation often arises as an emergent property of the holobiont—a consequence of shared resources, signalling interference, or immune trade-offs. Recognising these interactions shifts the focus from individual pathogens to the surrounding pathobiome that can constrain or sustain the success of obligate pathogens.

## What role does plant play in the interplay between microbes and obligate pathogens?

The plant is not a passive stage on which microbes act but an active orchestrator that shapes its pathobiome and, consequently, disease outcomes. By modulating immune signalling, metabolite release, and the chemical and structural, plants influence which microbes thrive and how they interact with obligate pathogens. This reciprocal feedback determines whether the pathobiome tilts toward disease suppression or facilitation.

Plants can actively recruit beneficial microbes in response to infection. Through the so-called “cry for help” strategy, plants alter root or leaf exudates and immune signals to recruit allies capable of suppressing pathogens. A recent study on *Arabidopsis* infected with *Hpa* showed the emergence of a reproducible “protective microbiome,” composed of commensals that suppress pathogen sporulation and enhance resistance to secondary pathogens [26]. Similarly, many beneficial microbes induce systemic resistance, effectively priming the plant for faster or stronger immune activation upon pathogen challenge. For example, rhizobacteria *Bacillus subtilis* can activate salicylic acid (SA)- and jasmonic acid-dependent pathways, increasing *PR-1* expression, and thereby reducing disease severity [27]. Endophytic fungi like *Serendipita indica* can also upregulate host antioxidant defences and cell wall fortification, making pathogen entry more difficult [28].

By contrast, some microbes—including obligate pathogens—actively suppress host immunity or remodel the microbiome to favour disease. *Albugo* spp. are a striking example: infection by *Albugo* suppresses host defences in *Arabidopsis*, permitting invasion by otherwise non-infective pathogens such as *Phytophthora infestans* [29,30]. Powdery mildew fungi can induce local susceptibility of invaded cells and secrete effectors that disrupt SA signalling and antimicrobial compound production, creating niches permissive to further colonisation and even promoting opportunities for genetic exchange between co-infecting strains [31–33]. Some pathogens suppress plant defences by directly manipulating the microbiota. *Albugo candida* secretes apoplastic proteins that selectively inhibit phyllosphere bacteria capable of triggering immunity [34]. As a result, beneficial commensals are removed, and opportunistic bacteria can proliferate. This strategy effectively disables a layer of microbiome-mediated resistance and creates a disease-favouring environment. Such microbiota manipulation is increasingly recognised as a sophisticated virulence strategy in fungal pathogenesis [3].

These processes show that plant immunity and physiology do more than react to single pathogens—they actively sculpt the microbial community, which in turn modulates disease outcomes. By modulating defence hormones, exudates, and signalling thresholds, the plant simultaneously influences both beneficial and deleterious microbial networks. Such plant-driven selection pressures not only determine the success of obligate pathogens but also parallel processes in hemibiotrophic and necrotrophic systems, where immune trade-offs and community shifts shape disease trajectories.

## Conclusion and perspectives

Obligate plant pathogens are embedded in a dense network of microbial interactions that can either hinder or facilitate their success. On one hand, microbial antagonists suppress pathogens through antibiosis, nutrient competition, niche pre-emption, or hyperparasitism, all natural mechanisms that limit colonisation and reproduction. On the other hand, some microbes support pathogens by providing essential metabolites, suppressing host immunity, or altering the microenvironment in favour of infection. These opposing interactions often co-occur, meaning that an obligate pathogen must navigate both support and suppression within a complex microbial community.

Recognising these dynamics opens new avenues for disease control. If microbiota can shift the balance between resistance and susceptibility, managing the microbial context becomes a viable strategy for disease control. Approaches like “pathobiome engineering” are emerging as tools in integrated pest management. Therefore, designing microbial consortia that reinforce holobiont resilience may enable more sustainable crop protection.

From an evolutionary perspective, obligate pathogens are particularly informative models because their survival depends on sustained coexistence with living hosts and associated microbiota. Their loss of metabolic autonomy, reliance on microbial metabolites, and secretion of effectors that suppress or restructure the microbiome all reflect deep coevolution within the pathobiome. Studying these systems can thus illustrate principles that extend beyond obligate pathogens—revealing how any plant-associated microbe, whether biotrophic, hemibiotrophic, or necrotrophic, is shaped by and contributes to the microbial ecology of disease.

Ultimately, moving beyond the classical two-player model of plant and pathogen or even three-player model of plant, pathogen and environment towards a holistic, microbiome-inclusive “disease diamond” will reshape our understanding of disease ecology (Fig 1). Yet, key questions remain. How do plants sense and selectively recruit protective microbes during infection? What roles do microbial effectors, secondary metabolites, and signalling exchanges play in shaping coexistence or competition within living tissues? And how can we bridge the gap between controlled *in vitro* studies and the complex, dynamic *in planta* environment? Addressing these challenges will link molecular and ecological perspectives, advancing both fundamental understanding and sustainable disease control.
